# Perspectives: Neonatal acute kidney injury (AKI) in low and middle income countries (LMIC)

**DOI:** 10.3389/fped.2022.870497

**Published:** 2022-08-31

**Authors:** Mignon I. McCulloch, Victoria M. Adabayeri, Selasie Goka, Tholang S. Khumalo, Nilesh Lala, Shannon Leahy, Nokukhanya Ngubane-Mwandla, Peter J. Nourse, Beatrice I. Nyann, Karen L. Petersen, Cecil S. Levy

**Affiliations:** ^1^Red Cross War Memorial Children’s Hospital, University of Cape Town, Cape Town, South Africa; ^2^Korle Bu Teaching Hospital, Accra, Ghana; ^3^Children’s Hospital of Philadelphia, University of Pennsylvania, Philadelphia, PA, United States; ^4^Nelson Mandela Children’s Hospital, University of the Witwatersrand, Johannesburg, South Africa; ^5^Chris Hani Baragwanath Academic Hospital, University of the Witwatersrand, Johannesburg, South Africa; ^6^Department of Paediatrics, University of Ghana Medical Centre, Accra, Ghana

**Keywords:** neonatal acute kidney injury, LMIC, neonatal KRT, neonatal peritoneal dialysis, conservative kidney management

## Abstract

Neonatal AKI (NAKI) remains a challenge in low- and middle-income countries (LMICs). In this perspective, we address issues of diagnosis and risk factors particular to less well-resourced regions. The conservative management pre-kidney replacement therapy (pre-KRT) is prioritized and challenges of KRT are described with improvised dialysis techniques also included. Special emphasis is placed on ethical and palliation principles.

## Introduction

Pediatric acute kidney injury (AKI) management is challenging in low- and middle-income countries (LMICs) where facilities for diagnosis and treatment including dialysis are limited. In view of this, prevention and early identification are important, specifically for neonatal acute kidney injury (NAKI) which is common in neonates and especially those admitted to neonatal intensive care units (NICUs). The kidney replacement therapy (KRT) in neonates has been associated with mortality rates of 17–24% as well as longer NICU or hospital stays (1). Newer generation KRT machines for neonates (*CARPEDIEM^®^* and NIDUS^®^) have been developed but are not easily accessible in many LMICs and thus there is a need in these regions for appropriate data on KRT in neonates and particularly accessible techniques such as peritoneal dialysis (PD).

### Diagnosis of neonatal acute kidney injury

Recognition of AKI (definition and diagnosis) has evolved to the KDIGO AKI definition as per Table 1 (2).

**TABLE 1:** Kidney disease: improving global outcomes (KDIGO) acute kidney injury (AKI) classification including neonatal modifications.

	Pediatric		Neonatal	
		
Stage	Serum creatinine	Urine output	Serum creatinine	Urine output^a^
1	1.5 to 1.9 times baseline OR ≥ 0.3 mg/dl increase*	<0.5 ml/kg/h for 6–12 h	≥ 0.3 rise within 48 h or ≥ 1.5–1.9 × rise from baseline (previous lowest value) within 7 days	≤ 1 ml/kg/h for 24 h
2	2.0–2.9 times baseline	<0.5 ml/kg/h for ≥ 12 h	2.0–2.9 times baseline	≤ 0.5 ml/kg/h for 24 h
3	3.0 times baseline OR Increase in serum creatinine to ≥ 4.0 mg/dl OR Initiation of renal replacement therapy OR In patients <18 years, decrease in eGFR to <35 ml/min per 1.73 m^2^	<0.3 ml/kg/h for ≥ 24 h OR Anuria for ≥ 12 h	≥ 3 × rise from baseline or serum creatinine ≥ 2.5 mg/dl or renal replacement therapy initiation	≤ 0.3 ml/kg/h for 24 h

^a^Urine output criteria utilized in the AWAKEN study. May also consider utilizing the pediatric urine output data for neonates if the granularity of data allows.

*Increase in SCr by X0.3 mg/dl within 48 h; or an Increase in SCr to X1.5 times baseline, which is known or presumed to have occurred within the prior 7 days. mg/dl, milligrams per deciliter; eGFR, estimated glomerular filtration rate; ml/min, milliliters per min; ml/k/h, milliliters per kilogram (2).

The neonatal definition should be used for children <120 days and the KDIGO pediatric definition (which is the same as the adult one) thereafter (3). Urine output (UO) is important for the assessment of neonatal AKI (4). However, AKI in the neonates is often non-oliguric (5), and the measurement of UO in neonates is limited by the availability of catheter sizes and the risk of catheter-associated urinary tract infections (UTIs) with sepsis (4).

### Limitations

Serum creatinine (SCr) is affected by age, sex, and muscle mass; changing in response to drugs, liver dysfunction, or fluid overload, and requires a baseline value for AKI diagnosis (6). In the neonatal period, maternal creatinine and maturational differences influence the SCr (3). Furthermore, preterm infants often have an initial rise in serum creatinine during the first few days of life before peaking and progressively declining (5). In term infants, serum creatinine gradually declines to reach a nadir by 2 weeks of age and even longer in preterm infants (3, 5). This could inherently be viewed as AKI, however, does not meet the current definition (2). In addition, there are significant delays in creatinine rise following an insult (48–72 h), and >50% function has to be lost before SCr will increase (3). In general, neonates have a low-glomerular filtration rate (GFR) at birth (even lower in premature infants) which gradually improves over the first few months of life, to reach adult values by the age of 2 years (3).

The application of newer biomarkers is a challenge in LMIC. A single-elevated SCr or oliguria should flag AKI to avoid delay in urgent treatment measures (6).

Low- and middle-income countries also experience delayed or unavailability of laboratory results in addition to lack of access to early markers of AKI; alternatively, attention to daily weights and meticulous recording of the absence of wet nappies may alert the clinician to AKI. Scoring systems such as the Renal Angina Index (RAI) become helpful in the early recognition of patients at risk for the development of AKI (2).

More recently, the “Neonatal AKI Risk Prediction Scoring” was devised as the “”STARZ (Sethi, Tibrewal, Agrawal, Raina, waZir)” Score analyzing the risk factors for AKI in neonates admitted to the NICU. This tool includes 10 variables (nine clinical and one laboratory) with a total score ranging from 0 to 100 and a cut-off score of 31.5, and has been successfully validated in a large multicenter cohort (7).

## Risk assessment and etiology

Risk assessment of NAKI focuses on perinatal and postnatal risk factor surveillance, and subsequently instituting primary prevention strategies (2), Table 2 below combines risk factors and common causes of NAKI (2, 8). In LMIC countries, dehydration and primary kidney disease are the commonest causes of NAKI but etiological data is lacking (2).

**TABLE 2:** Causes of neonatal AKI.

Combined risk factors	Prematurity
	Low birth weight
	Maternal risk factors predisposing to premature birth (pre-eclampsia, smoking and alcohol consumption)
	Maternal NSAID exposure
Pre-renal/ Functional AKI	Reduced renal perfusion
	Evaporative losses (premature neonates)
	Blood loss
	Gastrointestinal losses
	Reduced effective circulation/hypoxia
	Reduction in cardiac output
	Birth asphyxia
	Respiratory distress syndromes
	Critical congenital heart disease
	Cardiac surgery/ECMO
	Congenital heart block
	Sepsis syndromes
	Third spacing
	Nephrotoxic agents (therapeutic, traditional medications)
Intrinsic AKI	Tubular interstitial disease
	Ischaemic injury-hypoperfusion
	Asphyxia
	Sepsis syndromes
	Nephrotoxic agents (NSAIDS, Antibiotics)
	Renal vasculature disease
	Thrombosis (venous/arterial)
	Umbilical lines
	Glomerular, cystic disease
	Congenital nephrotic syndrome
	Renal cystic disease
	CAKUT (renal agenesis, dysplasia)
	Infections
	CKD
Post-renal AKI	Obstruction
	CAKUT (PUV, bilateral obstructive uropathy)
	Neurogenic bladder

In addition, nephrogenesis is incomplete in premature neonates thus immature glomeruli, impaired urinary concentrating ability, and autoregulation in neonates are additional NAKI risk factors. Unfortunately, the rates of premature deliveries are higher in LMIC with lower infant survival rates compared to high-income countries (9).

The 0by25 campaign, an International Society of Nephrology (ISN) awareness movement, states that no one should die of untreated AKI in low-resourced countries by 2025 by improving awareness, understanding, and policy implementation (10). Public health measures to provide clean water, endemic infections, environmental exposures, delayed recognition of AKI, accessibility to laboratory services, iatrogenic factors, and response to diagnosis are areas emphasized (10). The campaign describes the “5R’s,” namely; risk assessment, recognition, response, renal support, and rehabilitation (10).

### Preventing further injury

Prevention of neonatal AKI is important in LMIC where resources are limited. Nephrotoxic-AKI is common in hospitalized infants, particularly, infants with VLBW (2, 11). Surveillance, stewardship, and avoidance if possible, of nephrotoxic agents are important. It is known that the burden of neonatal sepsis is high in LMIC with potentially more exposure to nephrotoxic agents such as aminoglycosides being commonly used as first-line agents, however, this data is also lacking [Burden of Antibiotic Resistance in Neonates from Developing Societies (BARNARDS) study] (12).

Appropriate monitoring of serum drug levels of nephrotoxic drugs should be instituted (11) but this monitoring is frequently not available in less well-resourced countries in view of the cost of the drug level testing.

Urinary tract infections should be treated promptly to avoid the possibility of further kidney injury.

Secondary prevention strategies include early detection of neonatal AKI with standardized definitions and the use of biomarkers (2), as previously discussed. Tertiary prevention focuses on regular surveillance of long-term AKI complications, such as growth monitoring, blood pressure screening, and imaging to assess scarring of kidney parenchyma to ensure early investigation of possible evolving CKD.

## Management pre-kidney replacement therapy

The management of neonatal AKI begins with a thorough history and physical examination to determine risk factors, etiology, and severity of AKI (3). Volume status (hypo-, eu-, or hypervolemic) should be assessed by weight, vital signs, and mental and cardiorespiratory status. In addition, volume intake should be compared to output (urine, stool, and other losses). Urine output should be determined (oli-, non-oliguric, or polyuric) and electrolytes and renal bladder ultrasound obtained. Management of the neonate with AKI continues with achieving and maintaining euvolemia and safe electrolyte status and avoiding further kidney injury.

### Volume status

For hypovolemic patients, the fluid deficit should be repleted initially with bolus fluids and subsequent urine output reviewed. Updated Surviving Sepsis guidelines 2020 suggest smaller boluses of fluids at a time with volumes of 10–20 ml/kg/dose, especially in regions where intensive care facilities may not exist (13). Neonates who respond to initial fluid resuscitation can receive continuous fluids to support their maintenance needs and any additional losses. Patients who remain oliguric/anuric, should be fluid restricted to the minimum amount of volume (intravenous plus enteral) needed to prevent hypoglycemia. Hypervolemic patients will need volume restriction dependent on their state of oliguria.

Diuretics augment fluid removal in hypervolemic patients if the cardiovascular status is adversely affected. Frusemide increases urine output but does not alter the natural cause of AKI (3) however it is easier to manage an infant who passes urine than an anuric patient. For neonates, a trial of frusemide 1–2 mg/kg/dose may be given to induce diuresis together with theophylline (14, 15). If this is successful, a frusemide infusion of 0.2–1 mg/kg/h is useful in settings where KRT is not available. Neonates with polyuric AKI (such as infants with posterior urethral valves) are at risk of electrolyte losses and may require replacement.

### Electrolyte management

In general, oliguric patients with AKI should have restricted potassium intake (16). Specific therapy for hyperkalemia includes beta-adrenergic agonists *via* nebulizers or intravenous route, loop diuretics, intravenous calcium gluconate (10%), sodium bicarbonate, and cautious use of insulin and glucose therapy [monitoring for hypoglycemia to which neonates are particularly prone is important as even recent adult studies have cautioned against the use of insulin (17)] to shift extracellular potassium into the cells. Enteral cation exchange resins to remove potassium from the body have been implicated in colonic perforation and should be used to decant feeds instead and used only in extreme conditions (18).

Hypocalcemia should only be treated with calcium gluconate in severe cases or if the neonate is symptomatic. Phosphorus restriction, by giving breast milk or low-phosphorus formula, may be required in neonates with hyperphosphatemia, keeping in mind that neonates have a higher phosphate level at baseline. Oral phosphate binders may also be used.

Hyponatremia is often dilutional and free water intake restriction eventually corrects sodium levels in those situations. However, provision of sodium for partial slow correction is warranted in neonates with severe hyponatremia, those with neurological signs, and those with renal losses, with close monitoring.

Metabolic acidosis should be managed with Ringer’s Lactate when able, reserving sodium bicarbonate administration for severe cases (16).

## Acute kidney replacement therapy—peritoneal dialysis

The concept of “PD first” is the mainstay in many LMICs and also in neonatal AKI as venous access and machines relevant to neonates have historically been unavailable. PD catheters in form of Tenckhoff style catheters placed by surgeons or Seldinger placed catheters placed at the bedside have been successfully implemented (18). Simultaneous airway management and sterile techniques are essential with the creation of artificial ascites to prevent bowel damage prior to catheter insertion.

In situations where formal PD catheters are not available, Cook pigtail catheters, rigid stick catheters (Romsoms) and also improvised equipment such as central lines, chest drains, and nasogastric catheters can be used successfully as taught by the Saving Young Lives (SYLs) initiative.

More research is needed on the recommended prescription and catheters for low-birth weight babies and those used to date are summarized in this review by Burgmaier et al. (19). Prophylactic antibiotics by intravenous administration on insertion of a catheter or intraperitoneal technique prevent peritonitis and heparin can be used in the fluid bags.

Fluid volumes for neonatal AKI are 10–20 ml/kg/cycle with cycles consisting of fill (10–15 min), dwell (30–90 min), and drain (20–30 min) periods. In these small infants measuring devices such as buretrols for fluid administered are essential.

Fluid strengths vary from 1.5, 2.5, or 4.25% dextrose (or similar) and can be used as bicarbonate or lactate based. Locally prepared using buffered intravenous solutions such as Ringers Lactate with 50% dextrose added to form glucose solutions (see PDI guidelines) (17) mixed in a sterile manner have also been used successfully with low-infection rates.

Continuous flow PD (CFPD) using two PD catheters has been shown to significantly increase clearance and ultrafiltration during PD and research looking at gravity-assisted techniques show promise compared with attempting to do this with adapted automated machines (20).

Training teams of doctors and nurses together in improvisation techniques for KRT is part of the SYL strategy.

## Acute kidney replacement therapy–continuous kidney replacement therapy

Continuous kidney replacement therapy (CKRT) is increasingly used in neonates with oliguric AKI and associated volume overload or electrolyte derangements, or metabolic emergencies such as hyperammonemia. It is especially useful in neonates who are on ECMO, those who need very quick and high clearance rates (e.g., hyperammonemia) not achievable with PD and neonates with abdominal abnormalities that would not allow placement of a PD catheter or successful use of the peritoneal membrane and hemodynamically unstable patients (21). While CKRT has advantages, one needs to consider that vascular access is needed and carries risks such as infection, the circuit typically needs anticoagulation, the patient may be frequently exposed to blood if a blood prime is needed and, depending on the filter used, may develop bradykinin release syndrome (21, 22). Blood priming is less of an issue if the Cardio–Renal Pediatric Dialysis Emergency Machine (*CARPEDIEM^®^*), the Newcastle Infant Dialysis and Ultrafiltration System (NIDUS^®^) or the Aquadex^®^ machines are used (21, 22). While CKRT is currently unlikely to be a frequent option for patients in LMICs given the resources (technical, staffing, and cost) needed, we will review considerations further later.

Continuous kidney replacement therapy achieves fluid removal and clearance through diffusion (CVVHD), convection (CVVH), a combination of diffusion and convection (CVVHDF), and some adsorption (21). For the majority of neonates who are placed on CKRT, either of the options aforementioned is adequate to address the indications for acute dialysis. Double lumen vascular access, even as small as 5 Fr [Arrow ^®^ 5Fr 5 cm double lumen 18 and 20 g], 6 Fr [Powerhohn ^®^], or 6.5 Fr [Gamcath ^®^], is typically needed unless the *CARPEDIEM* ^®^ /NIDUS ^®^ is being used. Catheter sizes can be as low as 4 Fr, however, if the patient’s size would support a larger catheter size, this is preferable to allow for better flow rates and less clotting (21, 22).

For neonates in whom the extracorporeal volume is greater than 10% of their blood volume, a blood prime is recommended (22). Blood flow rates (Qb) should range from 3 to 5 ml/kg/min and the total dialysis dose around 20–25 ml/kg/h (2,000 ml/h/1.73 m^2^) keeping in mind that this dose may result in larger clearances for smaller neonates than they would for larger neonates. For anticoagulation, heparin or citrate (not licensed currently in neonates) are most commonly used. Most CKRT dialyzer membranes are replaced every 72 h but newer neonatal machines (e.g., *CARPEDIEM* ^®^) would need 24 hourly changes which are expensive in LMIC settings. Close monitoring of electrolytes is extremely important as is volume status.

## Affordability and improvisation when no equipment available

Where kidney replacement therapy (KRT) is indicated for neonatal AKI, very few families in LMICs are able to access centers equipped to perform this therapy (23).

Consequently, AKI is a death sentence for many children in these situations [Challenges of pediatric acute kidney injury in low-income and middle-income countries. Mignon McCulloch, Prasad Devarajan, on behalf of the International Pediatric Nephrology Association (IPNA guidelines)].

Many of these countries have large rural populations living large distances from medical centers. Transport infrastructure is also usually poor which compounds the problem. Consequently, children with AKI often have a delayed presentation with potentially avoidable complications (24).

Due to the unaffordable cost or unavailability of dialysis equipment, various improvisations have been used to perform PD. These include chest drains, nasogastric tubes, urinary catheters, and adult central venous lines, as a substitute for PD catheters as well as bedside prepared dialysis fluids adapted from intravenous fluids (25).

In many centers, only adult extracorporeal dialysis circuits are available and when used in small children may lead to fatal complications. In small babies, it is far better to use peritoneal dialysis than inappropriately sized hemodialysis equipment.

Drug and laboratory tests are mostly self-funded and thus are beyond the reach of the families. Clinicians are often required to make decisions based on experience and best guesses of prognosis without the aid of laboratory tests. Drug availability is also limited with the result that certain medications, especially, antibiotics are not selected according to guidelines, but choices are modified according to funds available.

## Chronic kidney replacement therapy

In the chronic KRT (cKRT), there is a need for frequent reviews at a tertiary facility. The need for transportation fares, and accommodation away from home pose a big challenge. Cost of drugs and laboratory tests are even more exorbitant than AKI management and often multidisciplinary teams, namely, dietitians, especially needed in neonatal CKD are non-existent (23).

In the case of chronic PD, storage space for PD fluids, running water, and dedicated sleeping space to perform PD are also limiting factors. The long-term goal for these children should be prepared for transplantation.

## Ethical issues around the provision of chronic kidney replacement therapy for neonates in low- and middle-income countries

The decision of whether to embark on a course of chronic kidney replacement therapy (cKRT) for a state-funded neonate in an LMIC requires careful consideration. Improvements in technology and outcomes (26–28) have led to an increase in the number of neonates started on cKRT (29), and the pediatric nephrologist in an LMIC may feel an obligation to commence cKRT for the neonate who needs dialysis. However, good outcomes in this population have only consistently been achieved by skilled teams working in resource-rich environments (30), and neonatal cKRT is not yet universally accepted as appropriate (31).

The discussion should quantify the expected quality of life and prognosis of the child, and address the expected quality of life for the family (32, 33). The physical, psychological, and social burden on the caregivers of children on cKRT is well-documented and the family social situation, distance, and means of transport from the dialysis center and reliability of electricity supply must all be considered (34).

The team should perform an honest appraisal of their ability to provide the requisite medical care (32), namely, the availability of equipment and medication, financial resources, level of medical and surgical expertise, and chances of future transplantation (24). Neonatal and infant dialysis requires lower fill volumes, shorter dwell times, and more frequent exchanges than older children (35). Most automated cyclers have a minimum fill volume of greater than 100 ml, and so the exchanges will initially have to be performed *via* a manual “buretrol” system (29). The neonate started on cKRT can expect to spend many months in an intensive care unit (ICU) setting until they are big enough to be managed on either an automated cycler or on a more manageable manual PD prescription.

Autonomy, beneficence, and non-maleficence all apply to issues in the neonatal ICU (36), but when it comes to commencing cKRT for the neonate in LMICs, the principle of distributive justice will dominate the others due to the critical shortage of neonatal and pediatric ICU beds in these areas (37, 38). ICU decision-making guidelines in LMICs emphasize the provision of equitable access and optimization of the overall benefit from the ICU resources, and so preference is usually given to children who have conditions with good outcomes, and who are predicted to have relatively short ICU stays (39).

What is unique about providing cKRT to a neonate is the long ICU stay that will be needed, especially, when morbidity can be significant, and long-term outcomes, including the prospect of transplantation, may well be guarded in this setting (30, 40). Perhaps commencing cKRT on neonates is not really a feasible option for most LMICs right now.

## Palliative care of the neonate with acute kidney injury

Palliative care provides multidisciplinary, holistic care to improve the quality of life in those with life-threatening or life-limiting illnesses, from diagnosis to bereavement (41, 42) including family counseling and symptom management (41). It is a goal for universal healthcare and should be provided by a specialized palliative team at all levels of care, including at home (41, 42). Palliative care is practiced worldwide, with a lag in pediatrics, especially in LMIC (43). It is not a part of the undergraduate curriculums, and overall knowledge of the goals of palliative care remains low, especially in junior staff (43).

Breaking significant news about a change in the course of illness, or management plan requires delivery in a manner considerate of the setting, people present, and actual information allowing questions and using mutual decision-making ensuring that the family’s anticipatory grief is not complicated by excess feelings of guilt or anger (44).

Advanced care planning is performed in a life-limiting illness, to redirect care toward comfort through symptom management, spiritual and psychosocial support. A reduction in invasive monitoring, imaging, and blood tests may be mutually agreed upon, even before the withdrawal of life-supporting therapies (45). Postnatal wards, intensive care units and also neonatal units can be utilized for end-of-life care.

Signs of discomfort should be identified and both non-pharmacological methods (swaddling and sucking) and analgesia provided according to availability and access (45, 46). Symptoms of uremia and electrolyte disturbances such as nausea, agitation, upper gastro-intestinal hemorrhage, and seizures should be managed appropriately, e.g., buccal midazolam for seizures, agitation, and distress.

Oliguric patients may develop symptoms of cardiac failure which may need reduction of intake as well as fentanyl for control of dyspnoea (45, 46). The use of dark bedding can assist in catastrophic bleeding.

Allowing the family an opportunity for memory making including photographs or cultural rituals has been shown to assist parents and siblings with their grief (45).

If possible to assist in planning, an estimated time as well as the place of death (tertiary referral center, a district hospital closer to home, a hospice, or at home) should be made in consultation with the family and entire team ensuring that it is in the best interest of the neonate and family, explaining the changes that may occur at and after death (44, 45). Neonatal units should be encouraged to develop protocols for end-of-life care that include control of symptoms, counseling structures as well as available community resources to support the families of those with AKI.

## Future goals

Education around neonatal AKI is an essential part of pediatric training together with advocacy for the development and funding of equipment suitable for these infants. Nephrology organizations, namely, IPNA, ISN, ISPD, and EuroPD with the Saving Young Lives program teaching adaptations and improvisation for PD, and also training fellowships, have come a long way in developing these services. However, advocacy at the hospital and government level in LMIC for funding of equipments is required to keep a focus on neonates with AKI.

## Data availability statement

The original contributions presented in this study are included in the article/supplementary material, further inquiries can be directed to the corresponding author.

## Author contributions

MM led the collaboration and planning of the manuscript, writing section and then combining all the sections, proofread, and did final edits and references. VA, SG, TK, NL, SL, NN-M, PN, BN, KP, and CL provided equally in writing separate sections with individual references and then also performed a final proofreading. It was an equal collaboration between a team of paeds nephrologists in LMIC. All authors contributed to the article and approved the submitted version.
